# Effect of elevated temperature and hydrocortisone addition on the proliferation of fibroblasts

**DOI:** 10.1007/s00418-024-02295-9

**Published:** 2024-05-27

**Authors:** Zuzana Pavlikova, Oldrich Zahradnicek, Anna Jelinek Michaelidesova, Jaromir Sramek, Marie Davidkova, Maria Hovorakova

**Affiliations:** 1 Institute of Histology and Embryology, First Faculty of Medicine, Charles University, Prague, Czech Republic; 2 Department of Anthropology and Human Genetics, Faculty of Science, Charles University, Prague, Czech Republic; 3 Department of Radiation Dosimetry, Nuclear Physics Institute, Czech Academy of Sciences, Prague, Czech Republic

**Keywords:** in vitro, Corticosteroids, Hyperthermia, Proliferation, Interaction, Fibroblasts

## Abstract

Hyperthermia along with hydrocortisone (HC) are proven teratogens that can negatively influence embryo development during early pregnancy. Proliferation of cells is one of the main developmental processes during the early embryogenesis. This study was focused on testing the effect of elevated temperature and HC addition on proliferation of cells in in vitro cultures. The V79-4 cell line was treated with HC and cultured in vitro at 37 °C or 39 °C, respectively. To reveal the effect of both factors, the proliferation of cells cultured under different conditions was evaluated using various approaches (colony formation assay, generation of growth curves, computation of doubling times, and mitotic index estimation). Our results indicate that a short-term exposure to elevated temperature slightly stimulates and a long-term exposure suppresses cell proliferation. However, HC (0.1 mg/ml) acts as a stimulator of cell proliferation. Interestingly, the interaction of HC and long-term elevated temperature (39 °C) exposure results in at least partial compensation of the negative impact of elevated temperature by HC addition and in higher proliferation if compared with cells cultured at 39 °C without addition of HC.

## Introduction

Early pregnancy is the period of an intensive cell proliferation, differentiation, and growth of embryonic tissues (Huppertz and Herrler 2005). These processes can be negatively influenced by external conditions that could finally result in a disrupted development of organs and organ systems. Cell proliferation, a highly controlled and regulated process, combines cell growth and cell division to produce daughter cells. A very high rate of cell proliferation is typical for early development of the human embryo.

Hyperthermia (Edwards et al. 1995; Suarez et al. 2004; Krausova and Peterka 2007), as well as distinct medicaments, such as hydrocortisone (HC; Peterka and Jelinek 1983; Edwards et al. 2003), can act as teratogens and can influence early morphogenetic processes, including cell proliferation. These negative factors may function independently or interact together during embryogenesis (Runner 1967).

Hyperthermia has been proven to cause embryonic death and malformations in a wide variety of species, including chicken, rats, rabbits, mice, hamsters, pigs, sheep, guinea pigs, monkeys (Edwards 1986), and humans (Graham 2020). Its negative effect has also been described in cell cultures. Reduced cell proliferation and viability have been observed as a consequence of high temperature exposure (Ibtisham et al. 2018; Siddiqui et al. 2020).

Hyperthermia as a factor influencing cell proliferation is also used for tumor treatment, usually in combination with chemotherapy and/or radiotherapy to enhance their therapeutic effect (Oei et al. 2015). It has been proven that hyperthermia (42 °C and 47 °C) treatment can initiate apoptosis of lung cancer cells in vitro. However, it can also generate sublines of these cells adapted to elevated temperature. The angiogenesis and proliferation potential of these cells has been shown to be induced by HIF-1a expression increased by hyperthermia (Wan and Wu 2016).

HC is a synthetic form of the steroid hormone cortisol synthesized in the zona fasciculata of the adrenal cortex. Owing to its anti-inflammatory and immunosuppressive properties, it is frequently used as a treatment in dermatology, rheumatology, immunology, and oncology. However, the teratogenic effect of synthetic glucocorticoids was proven by Fraser and Fainstat (1951), who induced cleft palate in offspring by injection of cortisone in pregnant female mice. The negative effect of glucocorticoid administration was also shown in other experimental animals, such as rabbits, hamsters, or chickens (Walker 1967; Shah and Chaudhry 1973; Peterka and Jelinek 1983). This effect was also described in humans, where the orofacial clefts were typical malformations induced by glucocorticoid administration (Park-Wyllie et al. 2000; Edwards et al. 2003). A negative effect of HC addition to culture media has also been observed in cell culture in vitro, with reduced population growth rates (Geiger et al. 1956) or cell death (Guichard et al. 2015).

Elevated temperature is the first symptom of numerous infections, and its occurrence during pregnancy is relatively frequent. However, its effect on proliferation, as well as the effect of its potential interaction with medication in pregnant women, for example, corticoids, is not fully understood. In the present study, we decided to test whether elevated temperature and the interaction of hyperthermia and HC addition could change the proliferation rate of cells in vitro. We cultured the fibroblasts of V79-4 cell line in vitro and tested the proliferation in cell populations under different conditions, such as elevated temperature and the addition of HC to the culture medium.

## Materials and methods

### Cell culture

Chinese hamster lung fibroblast V79-4 (RRID: CVCL_2796) cell line was obtained from the American Type Culture Collection (ATCC CCL-93). The cells were maintained as instructed by the distributor’s protocols, and they were grown and cultured in Dulbecco’s Modified Eagle’s medium (DMEM; Sigma-Aldrich) supplemented with heat-inactivated 10% fetal bovine serum (FBS; BIOSERA) and 0.5% antibiotics penicillin/streptomycin (Sigma-Aldrich) in TK25 tissue culture flasks for adherent cells (Techno Plastic Products AG) or MatTek glass-bottom dishes (MatTek).

The cells were cultured in six groups differing by their culture conditions (Table 1). The fibroblasts cultured at 37 °C, the temperature optimal for cell growth as recommend by the distributor’s protocol, represented the control group (C). The fibroblasts cultured at the elevated temperature of 39 °C represented experimental group 1 (E1). The fibroblasts in the other four experimental groups were treated with HC (VUAB Pharma a.s.) added directly in the culture medium at two different concentrations selected on the basis of our preliminary long-term experiments. The fibroblasts treated with HC at a concentration of 1 mg/ml and cultured at 37 °C represented experimental group 2 (E2). The fibroblasts treated with HC at a concentration of 0.1 mg/ml and cultured at 37 °C represented experimental group 3 (E3). The fibroblasts treated with HC at a concentration of 1 mg/ml and cultured at 39 °C represented experimental group 4 (E4). The fibroblasts treated with HC at a concentration of 0.1 mg/ml and cultured at 39 °C represented experimental group 5 (E5).

**Table 1:** Experimental groups for each method used in this study

Method	Tested group	Temperature [°C]	HC [mg/ml]
GC	GC-C	37	–
	GC-E1	39	–
	GC-E2	37	1
	GC-E3	37	0.1
	GC-E4	39	1
	GC-E5	39	0.1
MI	MI-C	37	–
	MI-E1	39	–
	MI-E3	37	0.1
	MI-E5	39	0.1
Number of colonies	Col-C	37	–
	Col-E1	39	–

The groups of in vitro experiments were sorted according to temperature and added HC concentration. *GC* growth curve, *HC* hydrocortisone, *MI* mitotic index

The in vitro cultures were exposed to different experimental conditions for 48 h (short-term exposure), or for more than 48 h (long-term exposure).

### The cell proliferation in the culture

The rate of cell proliferation was tested using two different approaches. The increasing number of cells in the cell population in in vitro cultures with time was evaluated using cell growth curves and doubling times (the doubling time of a population exhibiting exponential growth is the time required for a population to double). The proliferation of cells in culture was also determined on the basis of the estimation of mitotic index (MI).

### Cell growth curves and doubling times

The cells were cultured in TK25 tissue culture flasks (Techno Plastic Products AG) under different conditions. There were six tested groups according to the culture conditions: temperature and HC addition (GC-C and GC-E1–E5; see Table 1 above). The cultures were maintained for one up to 4 days. The cells were trypsinized and collected, and the number of cells in 1 ml of suspension was counted daily for each tested group using the Muse® Cell Analyzer (Luminex, RRID: SCR_020252) and Muse® Count & Viability Assay Kit (Luminex). The data obtained were used to construct the growth curves.

Doubling times (dt) of cell populations were calculated during the exponential/logarithmic phase of the growth according to the $N\, = \,N_{0} \, * \,2^{{\left( {t/dt} \right)}}$ formula (*N*_*0*_ is the number of seeded cells, *N* is the number of cells after time *t* from seeding), using open-source software Gnuplot 5.0 patchlevel 6 (RRID: SCR_008619). Statistical differences between dts of experimental groups were evaluated using the analysis of variance (ANOVA) test. The differences were considered statistically significant if *p* < 0.05.

### Analysis of proliferation using mitotic index estimation based on fluorescent labeling of mitotic cells and confocal imaging

V79-4 cells were cultured in MatTek glass-bottom dishes (MatTek) under different conditions. Thus, there were four tested groups according to temperature and HC (at a concentration of 0.1 mg/ml) addition (MI-C, MI-E1, MI-E3, and MI-E5; see Table 1 above).

Each dish was seeded with 500 cells and cultured in vitro for 4 days. Then, the fibroblasts were fixed in 4% paraformaldehyde (PFA) and stained using a standard immunofluorescence protocol. As a marker of cell-proliferation rabbit antibody Ki67 (Abcam Cat# ab15580, RRID: AB_443209) was used, followed by donkey anti-rabbit immunoglobulin G (IgG) Alexa Fluor® 594 secondary antibody (Molecular Probes, Invitrogen Cat# A-21207, RRID: AB_141637). 4′,6-Diamidino-2-phenylindole (DAPI; Sigma-Aldrich, Cat# 268298) was used for nuclei counterstaining. Images of cell colonies were obtained with the confocal system FluoView™ FV1000 on an upright BX61 Microscope (Olympus Czech Group Ltd., Prague, Czech Republic; RRID: SCR_020337) using Olympus FluoView Viewer software (Ver.4.2b; RRID: SCR_024433). Imaging was performed using an Olympus UPlanSApo 20 × dry objective (NA 0,75; WD 0,6), format 1024 × 1024 pixels, and sequential scanning mode. The appropriate filter sets were used to properly capture Alexa Fluor® 594 that was excited with a laser wavelength of 543 nm. For DAPI excitation, a laser wavelength of 405 nm was used. The ratio of proliferating cells to the total number of cells of each colony in the sample was evaluated for each group using developed evaluation application (see below). The differences between groups were statistically analyzed using the Kruskal–Wallis test followed by a post-hoc Mann–Whitney *U* test.

The MI was estimated as the ratio of the overall area of particles in the red channel (red = Ki67 positive nuclei) to the overall area of particles in the gray-level channel (gray = all nuclei) in confocal micrographs. An application to count the MI from micrographs was developed. A short script written in Python 3 (RRID: SCR_008394) using methods from the library OpenCV2 for the estimation of the mitotic rate was used. The micrographs were preprocessed using adaptive contrast filters and morphological denoising to remove small parasitic signals.

In the next step, the image was split into two gray-level images: one was the transformation to the gray-level image of the original image, and the second was the red channel of the original image. The first image represented the signal of all nuclei, and the second image represented the signal of mitotic nuclei. The function findContours detected particles in both images. Particles of areas up to 30 pixels were classified as artifacts and removed from the analysis. Particles of areas greater than 30 pixels represented one or occasionally more nuclei. Therefore, areas of these particles were used for the main estimation of the MI. The MI was estimated as the ratio of the overall area of particles in the red channel to the overall area of particles in the gray-level channel.

### Colony formation assay

The effect of different temperatures on the proliferation of cells was also evaluated using colony formation assay for six independent experiments. In three independent experiments, the six-well plates were seeded with 100 or 150 V79-4 cells in each well of the plate, respectively. There were two six-well plates for each experiment cultured under different conditions. The control group (Col-C) consisted of cells cultured at 37 °C. The experimental group was cultured at 39 °C (Col-E1). After 6 days, the colonies of fibroblasts were fixed in 4% PFA, stained with crystal violet (Lach-Ner), and finally counted. The number of colonies was counted three times for each well and averaged. The differences in numbers of colonies between both groups were statistically analyzed using the Mann–Whitney *U* test or two-sample *t*-test, respectively. The differences were considered statistically significant if *p* < 0.05.

## Results

**Fig. 1:**
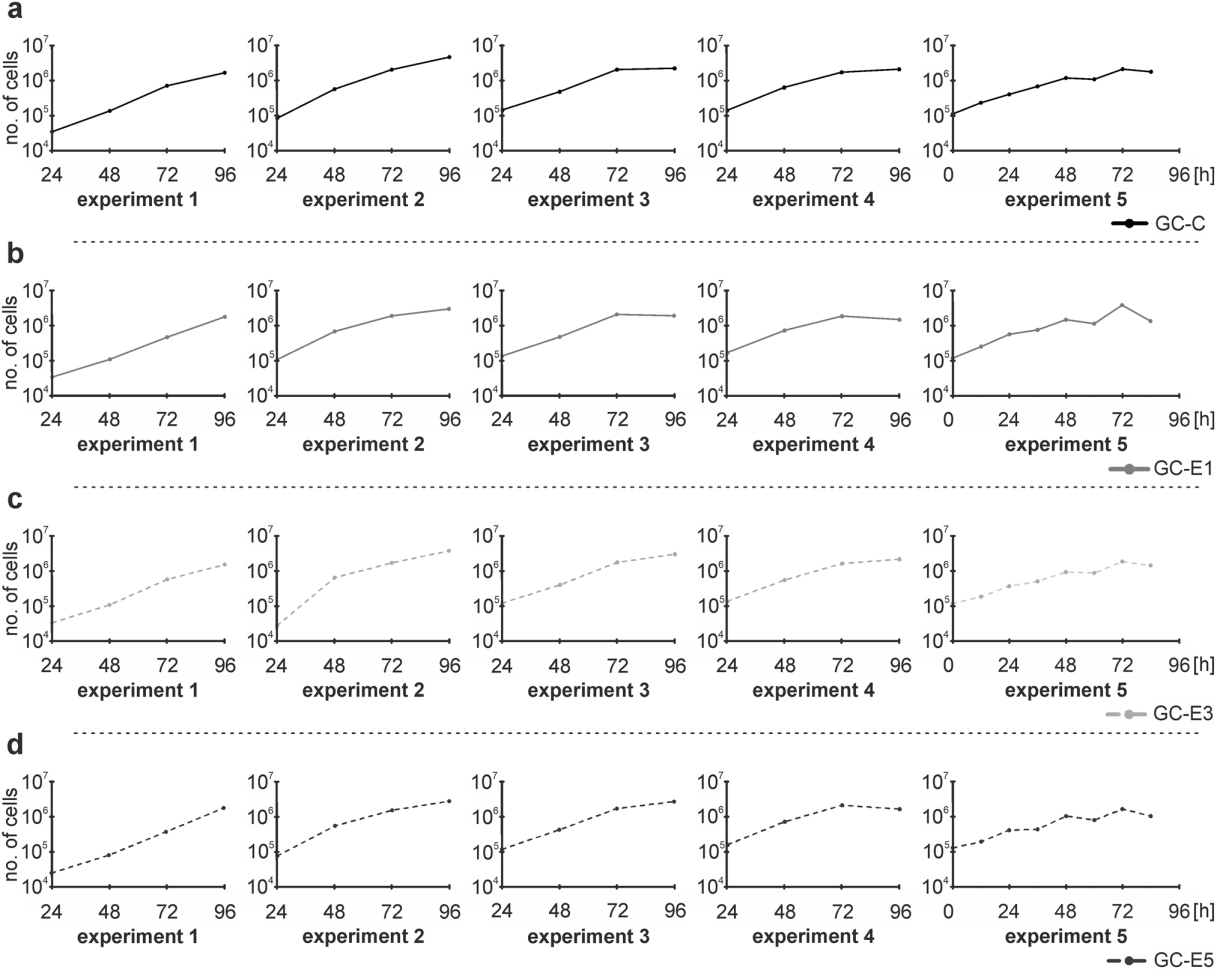
The growth curves for cells cultured under different conditions in five independent experiments. A logarithmic transformation of the growth curves was performed.The growth curves are shown for cells cultured: **a** at 37 °C (GC-C), **b** at 39 °C (GC-E1), **c** at 37 °C with addition of HC (GC-E3), and **d** at 39 °C with addition of HC (GC-E5). The *x*-axis shows time in hours, and the *y*-axis shows the number of cells

To evaluate the growth of the cell cultures under different conditions, the growth curves showing the change in the number of cells in a culture at different times were constructed for each tested group (GC-C and GC-E1–E5) in five independent experiments (Fig. 1). Dts were determined from the exponential growth phases to show the time in which the cells in the culture doubled their number.

### The proliferation of cells evinced a slight increase with elevated temperature

**Fig. 2:**
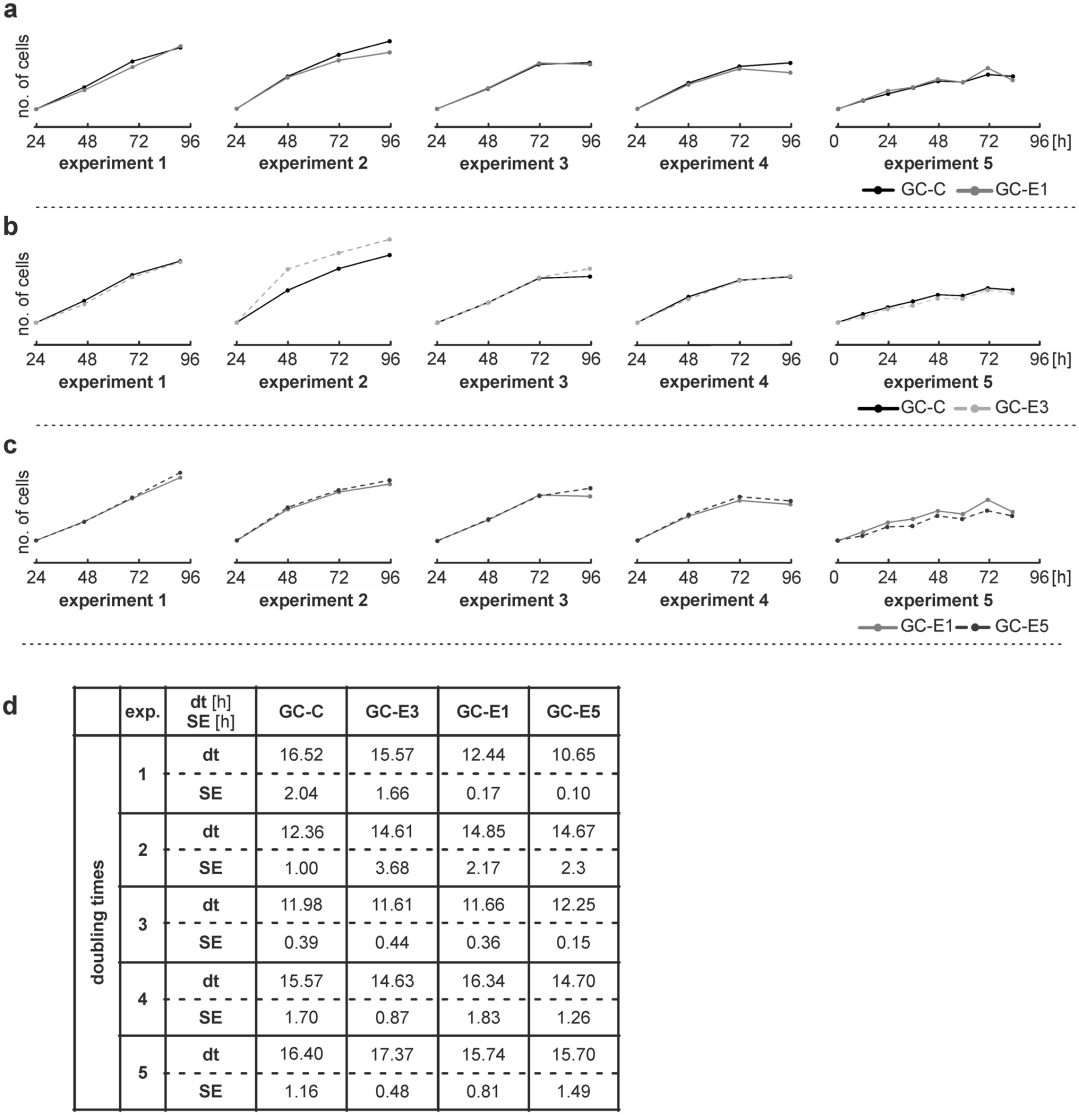
Growth curves and doubling times (dt) of cells cultured under different conditions. A logarithmic transformation of growth curves was performed. **a** The growth curves for cells cultured at 37 °C (GC-C) and at 39 °C (GC-E1) for five independent experiments indicated slightly higher proliferation of cells cultured at 39 °C (GC-E1) in experiments 3 and 5 compared with the control group cultured at 37 °C (GC-C). **b** The growth curves for cells cultured at 37 °C with addition of HC (GC-E3) for five independent experiments did not reveal any substantial changes compared with the control group without the addition of HC (GC-C). **c** The growth curves for cells cultured at 39 °C without the addition of HC (GC-E1) and at 39 °C with addition of HC (GC-E5) for five independent experiments exhibited slightly increased proliferation of cells cultured with HC addition and at 39 °C (GC-E5) in experiments 1, 2, and 4 compared with cells cultured at 39 °C (GC-E1) without the addition of HC. **d** The dts in hours (h) are shown for each tested group (GC-C, GC-E3, GC-E1, and GC-E5) of five independent experiments (exp.) with standard errors (SE; h). The ANOVA test was used to test differences between tested groups across five independent experiments. The differences between the groups were not significant (*p* = 8.29 × 10^−01^)

The obtained growth curves (Fig. 2) indicated slightly higher proliferation of cells cultured at 39 °C (GC-E1) compared with the control group cultured at 37 °C (GC-C; Fig. 2a). To analyze this slightly positive effect of elevated temperature on proliferation, we calculated dts, which showed the time needed for the population of cells to double in number. In accordance with observed differences in the growth curves, the dts for cells cultured at 39 °C (GC-E1) were slightly lower in three out of five independent experiments compared with cells cultured at 37 °C (GC-C; Fig. 2d). However, the differences between the groups tested using the ANOVA test were not significant.

### Optimalization of HC concentration for further testing

The effect of two different concentrations of HC in DMEM (1 mg/ml and 0.1 mg/ml) at optimal (37 °C) and elevated (39 °C) temperatures on the cell proliferation was tested using growth curves. They showed suppressed cell population growth at a higher concentration of HC (1 mg/ml; GC-E2 and GC-E4) at both temperatures tested. The negative impact of a higher concentration of HC was also proven by MI analysis and colony formation assay. In both cases, no colonies were detectable at the end of the experiment. Thus, the number of colonies and MI could not be estimated. According to these results, the 0.1 mg/ml concentration of HC was used for further experiments.

### HC at a concentration of 0.1 mg/ml stimulated cell population growth

The effect of HC (0.1 mg/ml) at optimal (37 °C) and elevated (39 °C) temperatures on cell proliferation was tested using growth curves and dts (Fig. 2). The growth curves for cells cultured with the addition of HC at 39 °C (GC-E5) documented slightly increased proliferation in several independent experiments compared with cells cultured at 39 °C (GC-E1) without the addition of HC (Fig. 2c). However, the effect of HC addition at 37 °C was not evident (Fig. 2b).

To confirm the slightly positive effect of HC on proliferation observed at 39 °C, we calculated dts (the time needed to double the cell number) for cell populations (Fig. 2d). In four out of five independent experiments performed, the cells cultured with the addition of HC at 39 °C (GC-5) had lower dts than cells cultured without the addition of HC at 39 °C (GC-E1). Nevertheless, the dts were lower in three out of five experiments for cells cultured with the addition of HC at 37 °C (GC-E3) compared with the control group without the addition of HC (GC-C). These results showed that HC addition (at a concentration of 0.1 mg/ml) positively influenced proliferation by reducing the time needed for doubling the number of cells in the population, especially at 39 °C. However, the ANOVA test showed no significant differences between tested groups (*p* = 8.29 × 10^−01^).

### Elevated temperature negatively influenced MI of the cells

**Fig. 3:**
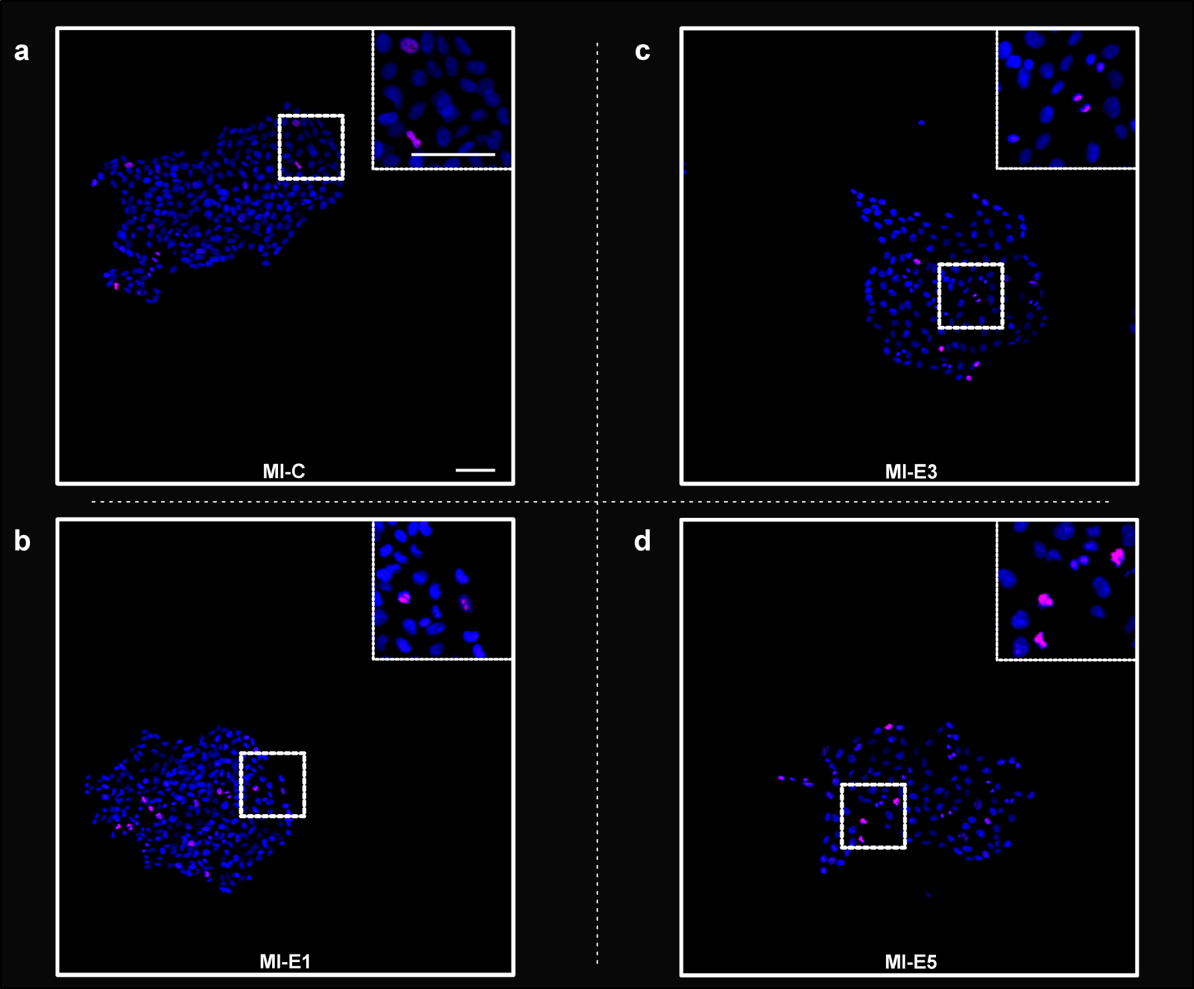
Representative microphotographs of cell colonies cultured under different conditions used to estimate MI. **a** A colony of cells cultured at 37 °C (MI-C), **b** a colony of cells cultured at 39 °C (MI-E1), **c** a colony of cells cultured at 37 °C with the addition of HC (MI-E3), and **d** a colony of cells cultured at 39 °C with the addition of HC (MI-E5) are shown. As a marker of cell-proliferation, antibody Ki67 (red) was used. DAPI (blue) was used for nuclei counterstaining. The detail in the insert in the right upper corner always represents the area delimited by the white dotted square in the original image. Scale bars: 50 µm

**Fig. 4:**
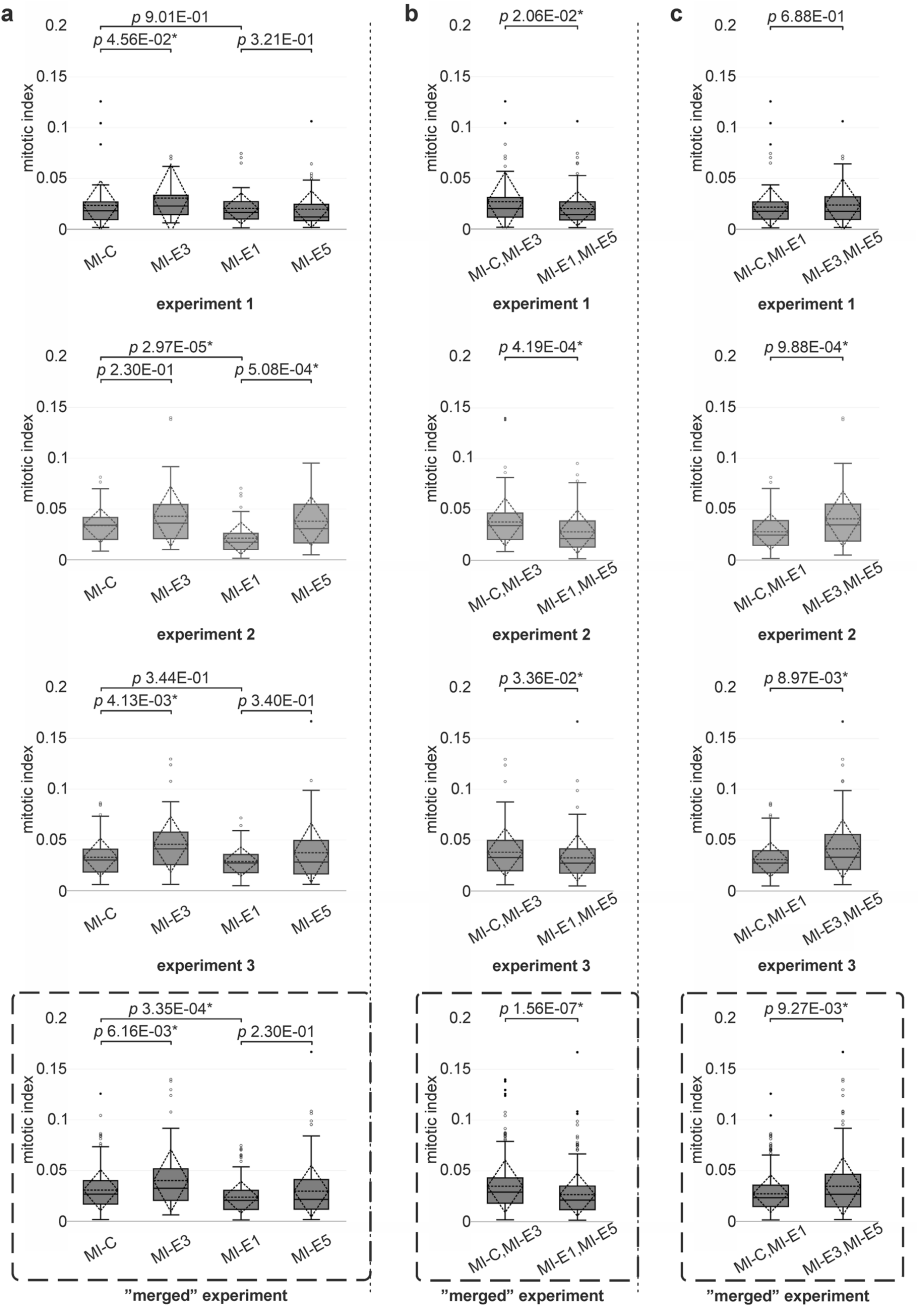
MIs estimated for colonies of cells cultured under different conditions. MI for each tested group was visualized using box and whisker plots (standard deviation—dashed rhombus, mean—dashed horizontal line, median—solid line). **a** The MIs estimated for colonies of cells cultured at 37 °C or 39 °C and without or with the addition of HC (MI-C, MI-E3, MI-E1, and MI-E5) for three independent experiments as well as for the “merged” experiment showed a negative effect of elevated temperature (MI-E1) compared with the control group (MI-C) and a positive effect of HC addition on cell proliferation in both tested temperatures (MI-E3 and MI-E5) compared with cells cultured without the addition of HC (MI-C and MI-E1). The Kruskal–Wallis test (*p* = 1.68 × 10^−02^*, *p* = 6.49 × 10^−06^*, *p* = 4.55 × 10^−03^*, and *p* = 8.17E × 10^−08^*) and post-hoc Mann–Whitney *U* test were used to analyze the MIs. **b** The MIs of cells cultured at 37 °C or 39 °C for three independent experiments and also for the “merged” experiment evinced significantly lower MIs for cells cultured at 39 °C without or with the addition of HC (MI-E1 combined with MI-E5) compared with the groups cultured at 37 °C without or with the addition of HC (MI-C combined with MI-E3). **c** The MIs of cells cultured without or with addition of HC for three independent experiments and also for the “merged” experiment evinced significantly lower MIs for cells cultured without HC (MI-C combined with MI-E1) compared with the groups cultured with addition of HC (MI-E3 combined with MI-E5). The significance of the calculated *p*-values is labelled with an asterisk (*)

Mitotic index (MI) was estimated for colonies of four tested groups differing in culture conditions. The cells were cultured at 37 °C (MI-C), at 39 °C (MI-E1), at 37 °C with the addition of 0.1 mg/ml HC to the culture media (MI-E3), and at 39 °C with the addition of 0.1 mg/ml HC to the culture media (MI-E5) in three independent experiments (Figs. 3, 4). The differences between tested groups were also evaluated in one common set merging the data from all three independent experiments (hereafter referred to as the “merged” experiment).

The normality of the data was verified using the Shapiro–Wilk test. In all tested groups across independent experiments, a significant deviation from the normal distribution was detected, and it could be assumed that the data were not normally distributed. Thus, non-parametric tests were used for further evaluation. The Levene’s test was performed to test the homogeneity of variance for each tested group (MI-C, MI-E1, MI-E3, and MI-E5) across three independent experiments. Statistically significant differences were detected only within groups MI-E5 (39 °C, 0.1 mg/ml HC; *p* = 0.03). Furthermore, the differences between groups were tested using the Kruskal–Wallis test followed by post-hoc Mann–Whitney *U* test for comparison of all four tested groups. When only two groups were compared, the Mann–Whitney *U* test was used. The elevated temperature had a negative impact on the cell proliferation (Fig. 4a). Even still, the differences between cells cultured at 37 °C (MI-C) compared with cells cultured at 39 °C (MI-E1) were statistically significant in only one of the independent experiments (*p* = 2.97 × 10^−05^).

To prove the negative effect of elevated temperature on cell proliferation, the MIs of cells cultured at 37 °C (group 37 °C: MI-C combined with MI-E3) were compared with MIs of cells cultured at 39 °C without or with HC addition (group 39 °C: MI-E1 combined with MI-E5). Cells cultured at 39 °C (MI-E1 combined with MI-E5) had significantly lower MIs than cells cultured at 37 °C (MI-C combined with MI-E3) in all three independent experiments as well as in the “merged” experiment (Fig. 4b), proving that elevated temperature alone had a negative effect on cell proliferation in culture.

### The addition of HC positively stimulated proliferation of cultured cells

The addition of HC to culture media had a positive effect on cell proliferation (Figs. 3, 4). The differences between groups of cells cultured at 37 °C (MI-C) compared with cells cultured with addition of HC at 37 °C (MI-E3) were statistically significant in two independent experiments and also in the “merged” experiment (Fig. 4a). The positive impact of HC addition to culture media was also observed at 39 °C; however, the differences were not as evident as at 37 °C. The differences between cells cultured at 39 °C (MI-E1) compared with cells cultured with the addition of HC at an elevated temperature (MI-E5) were statistically significant only in one independent experiment (Fig. 4a).

The positive effect of HC on proliferation was also proven by comparison of the two groups cultured without HC addition (MI-C combined with MI-E1) with the two groups cultured with HC addition (MI-E3 combined with MI-E5) for all three independent experiments. The differences between groups cultured without HC addition (MI-C combined with MI-E1) and with HC addition (MI-E3 combined with MI-E5) were statistically significant in two independent experiments as well as in the “merged” experiment (Fig. 4c).

To summarize the effect of HC addition to culture media on MIs, HC addition increased cell proliferation at both tested temperatures.

### Elevated temperature reduced the number of colonies

To determine the effect of temperature on cell proliferation, the number of colonies was counted in six independent experiments. The differences between the tested groups were evaluated for each independent experiment and in two sets comprising the merged data from three independent experiments, where 100 cells or 150 cells were initially seeded, respectively (hereafter referred to as the “merged” experiment).

**Fig. 5:**
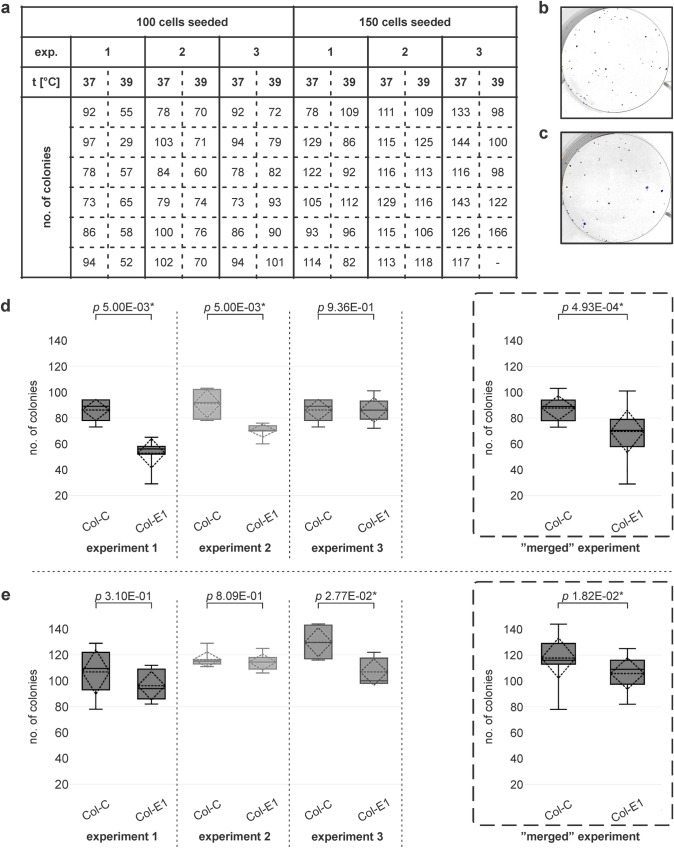
The number of colonies for each tested group cultured after seeding (**a**) 100 and 150 cells per dish, respectively. The representative image of colonies of cells grown (**b**) at the dish at 37 °C (Col-C) and (**c**) at 39 °C (Col-E1) is shown. The numbers of colonies for Col-C and Col-E1 formed after (**d**) 100 and (**e**) 150 cells were seeded on dishes, respectively, are visualized using box plots, where standard deviation is displayed as a dashed rhombus, along with the mean (dashed horizontal line) and median (solid line) values. The box and whisker plots showed that the average number of colonies was higher at 37 °C (Col-C) compared with 39 °C (Col-E1). Significance of *p*-values of the Mann–Whitney *U* tests or two sample *t*-tests (for “merged” experiments) is labelled using an asterisk (*). *exp* experiment, *t* temperature

Two tested groups of six independent experiments cultured at different temperatures were used to analyze the number of colonies (Fig. 5a). The first group consisted of cells cultured at 37 °C and served as a control (Col-C). The other group was cultured at 39 °C (Col-E1). In five out of six independent experiments, there was a higher average number of colonies grown at 37 °C (Col-C) compared with those grown at 39 °C (Col-E1; Fig. 5b, c). The obtained data were analyzed using the Mann–Whitney *U* test. The differences between groups (Col-C and Col-E1) were statistically significant in two out of three independent experiments where 100 cells were initially seeded (Fig. 5d) and in one out of three independent experiments where 150 were seeded (Fig. 5e).

The complex analysis was performed for all three experiments where 100 cells and 150 cells were seeded, respectively. The differences between average numbers of colonies cultured at 37 °C (Col-C) and at 39 °C (Col-E1) were statistically significant in both cases, in the “merged” experiment with 100 cells seeded (*p* = 4.93 × 10^−04^) and the “merged” experiment with 150 cells seeded (*p* = 1.82 × 10^−02^), respectively (Fig. 5d, e). The average number of colonies in the group Col-C (37 °C) was significantly higher compared with the group Col-E1 (39 °C).

## Discussion

It has been shown previously that hyperthermia and HC may negatively impact the development of the embryo. These negative factors may act independently or interact together. According to the resulting effect, four types of interactions are recognized: nil effect (the lack of any observable interaction when two factors are used together), interference (protective), additive, and potentiation (Runner 1967). In our study, the cultures of fibroblasts were treated with HC added to the culture medium and cultured in vitro under different conditions to test the effect of elevated temperature and HC, as well as the effect of their interaction on cell proliferation.

Our results obtained using different approaches showed that a HC concentration of 0.1 mg/ml in culture media had a stimulating effect on the fibroblast proliferation. The positive effect of HC on cell proliferation was also proven by Arpels et al. (1964). They found that HC addition improved the population growth of several cell lines in a cell culture (lines HEp 1 and RPMI-41). HC at a concentration of 0.0025–0.01 mg/ml substantially extended the length of good growth, indicating that the cultures remained in excellent condition without refeeding the cultures with fresh medium for a longer time than all control cultures (Arpels et al.^1964^). Prolonged lifespan, shortened generation time, and higher plating efficiency were observed after the addition of cortisone at a concentration of 0.0025 mg/ml to culture medium (Macieira-Coelho 1966). Interestingly, the results of the two studies mentioned above showed a positive effect of HC addition on cell population growth at HC concentrations lower than one order in magnitude compared with our results. These very low concentrations seem to have an impact on cell proliferation over a longer period of time. However, an inhibitory effect of HC on the growth of fibroblasts obtained from cardiac explants has been reported. Inhibition of growth was demonstrated at HC concentrations as low as 0.2 mg/ml (Grossfeld and Ragan 1954). A similar effect of HC addition was also described in adult mouse, rabbit, and chicken fibroblasts that were exposed to cortisone at concentrations of 0.01–0.1 mg/ml. These concentrations had a significant inhibitory effect on cell population growth rate. Surprisingly, these concentrations of cortisone had no effect on the growth of embryonic fibroblasts, nor on the growth of adult human epithelial cells in vitro (Geiger et al. 1956). No effect of HC at a concentration of 0.2 mg/ml on cell population growth was observed in gastric and intestinal epithelial cells (Grossfeld and Ragan 1954). However, a negative effect of HC at concentrations of 0.01 mg, 0.1 mg, and 1 mg in vitro was proven using human cell lines, specifically three epithelial cell lines and one cell line derived from the connective tissue. In general, the epithelial cell lines required longer exposure to HC in contrast to cell lines derived from normal infant’s foreskin before any cytotoxic effect was detected (Kline et al. 1957).

The negative impact of HC on cell proliferation proven by studies mentioned above was caused by HC at concentrations ranging from 0.01 mg/ml to 1 mg/ml. However, our data showed that HC’s effect on proliferation is concentration specific. In our experiments, HC at a concentration of 1 mg/ml had an inhibitory effect on cell proliferation in contrast to HC at a concentration of 0.1 mg/ml, which had a stimulating effect on V79-4 cell proliferation. It seems that the effect could also be cell population (cell line) dependent. The studies of Grossfeld and Ragan (1954), Geiger et al. (1956), and Kline et al. (1957) also showed the inhibition of population growth at a concentration of 0.2 mg/ml and lower, which was proliferation-stimulating in our case. The differences could also be related to different initial cell numbers in the culture, different duration of the experimental manipulation of cell cultures, different culture conditions, or different effects of HC during short- and long-term experiments.

Interestingly, it has been shown that glucocorticoids (including HC) also evince cytotoxic effect (Guichard et al. 2015) and antiproliferative properties (Hammer et al. 2004).

It has been proven that glucocorticoids trigger cell death in certain cell types, such as monocytes (Schmidt et al. 1999), osteoblasts, and osteocytes (O’Brien et al. 2004) or tumor cells (Yamaguchi et al. 1999). The ability of glucocorticoids to inhibit cell growth has been used for decades in the treatment of childhood acute lymphoblastic leukemia (Gaynon and Lustig 1995).

Our results indicated that the effect of HC on the number of cells in the cell culture in our experiments was positive, even if not significant in all cases. In these cases, the increased cell death could be one of the factors playing a role in the final impact on cell culture growth since it has been shown that HC can induce apoptosis in distinct cell types, such as monocytes (Schmidt et al. 1999), tumor cells (Ikramova 2020), or keratinocytes (Guichard et al. 2015). However, the effect of HC on apoptosis seems to also be related to the cell line, growth of cells in suspension or monolayer, duration of HC treatment, and the HC dose used, and similarly to the effect of HC addition on cell proliferation.

Temperature has been found to have a great impact on biological processes in general. Cell cultures in vitro are valuable systems ideal for testing its impacts since the temperature can be controlled relatively easily.

Our results showed that long-term exposure to a temperature of 39 °C had a negative impact on fibroblast proliferation (Figs. 4, 5). However, many studies documented that short-term exposure to mild heat stress can positively regulate cell proliferation and viability. The mild heat stress with optimal temperatures between 39 °C and 40 °C supported proliferation and osteogenic differentiation of dental follicle stem cells (DFSCs; Rezai Rad et al. 2013). The periodic mild exposure of mesenchymal stem cells (MSCs) to the temperature of 41 °C was beneficial for cell viability, proliferation, and differentiation, and it led to the delayed senescence of the cells (Choudhery et al. 2015). Increased proliferation and enhanced neuronal proliferation after mild heat exposure was observed in the neural stem/progenitor cells (NSCs/NPCs) exposed to the temperature of 38.5 °C for 4 days (Hossain et al. 2017). Heat stress of 41 °C significantly stimulated viability of broiler fibroblasts within a short exposure time (24 h) unlike long-term exposure (for 3 days), which caused cell cycle arrest, induced apoptosis, and reduced cell viability (Siddiqui et al. 2020). Similar results were shown for chicken embryonic fibroblasts (CEFs) that were cultured at different temperatures (37 °C and 40–44 °C) for 6, 12, and 24 h, respectively. CEFs cultured at 41 °C presented significantly higher cell viability at distinct time points compared with the control. However, the cell proliferation and viability at temperatures above 41 °C were decreased in a time-dependent manner (Ibtisham et al. 2018). Results of these abovementioned studies demonstrated the beneficial effect of mild heat exposure for a short time on proliferation and viability of the cells. These findings are in accordance with our experiments revealing the positive effect of elevated temperature within first 48 h of incubation in case of doubling time evaluation. However, our data based on exposure for longer than 48 h showed a negative impact of elevated temperature on cell proliferation (colony formation assay and MIs). The negative impact of a long-term heat exposure has been shown to cause decreased cell proliferation and viability (Ibtisham et al. 2018; Siddiqui et al. 2020). The negative effect of high temperature on cell proliferation was shown in cardiac muscle tissue fragments of chick embryos that were exposed to temperatures of 5 °C, 12 °C, 20 °C, 30 °C, 39 °C, and 45 °C in vitro. It was observed that growth rate was highest at 39 °C and lowest at 45 °C. The tissue fragments kept at temperatures of 5 °C, 12 °C, and 20 °C did not multiply at all (Nemoto 1929). The prolonged exposure to supranormal temperatures of 42 °C and 44 °C on the growth of chick osteoblast in vitro did not show any lethal effect. However, the temperatures higher than 44 °C suppressed the cell population growth, and the death of cultures was observed after 105 min exposure to 47 °C, after 6 min exposure to 50 °C, and after 3.5 min exposure to 52 °C (Pincus and Fischer 1931). Interestingly, the inhibitory effect of supranormal temperatures in the range of 40–43 °C has been widely used to prevent tumor cells from proliferation during cancer treatment (Field and Bleehen 1979; Wust et al. 2002).

Based on these controversial observations, the effect of the elevated temperature is quite ambiguous. The response to heat stress may depend on cell type/line, developmental stage, temperature dose, and exposure time, similarly to the HC effect. The differences in the heat sensitivity of various cell lines were described. These differences could be related to the inherent variances between cell lines, growth of cells in a suspension or in a monolayer, observation of cells in vivo or in vitro, the tumorigenicity of cell lines, or the stage of the cell cycle and growth phase. It was shown that Chinese hamster ovary cells in late S-phase were more sensitive to heat stress than cells in early S-phase and in mitosis. Cells in G_1_ or G_2_-phases were the least sensitive to heat (Bhuyan et al. 1977). Similar results have been shown in S-phase and in mitotic cells, which were revealed as the most sensitive cell cycle phases to a heat shock of 43.5–46.5 °C. The cells in mitosis exposed to the heat shock failed to finish cytokinesis and became tetraploid (Westra and Dewey 1971). It was also proven that the cells grown in a suspension were slightly less sensitive to a temperature of 43 °C than the cells grown in a monolayer (Bhuyan et al. 1977).

Even though there are many variables that influence the effect of elevated temperature on cell proliferation and viability, it was shown that, although apoptosis and cell cycle arrest have been demonstrated in numerous studies, fever-range elevation of temperature or mild heat stress may positively regulate proliferation and differentiation of the cells via modulation of physical properties and activities of several regulatory proteins (Park et al. 2005). Interestingly, in pregnant women, basal body temperature (BBT) has been shown to remain elevated throughout approximately the first 4 months of a pregnancy (Zuck 1938; Buxton and Atkinson 1948; Siegler 1955). This slightly increased BBT could positively influence cell proliferation, which is one of the embryogenetic processes running during the first trimester of pregnancy.

Our results showed that HC at a concentration of 0.1 mg/ml exhibited a stimulating effect on cell proliferation. According to Runner’s (1967) four types of responses to concomitantly administrated substances, we could postulate that the main type of interaction between HC and elevated temperature during long-term exposure was protective (interference). Protective interaction means that the effect of one substance reduces the effect of another substance. In our case, the situation was slightly different because, unlike Runner (1967), one teratogen was of chemical and one of physical nature.

A deficit of cells in the brain has been observed in newborn guinea pigs prenatally exposed to short-term hyperthermia of 42.0–42.5 °C for 1 h on day 21 of gestation. The deficit of brain cells was related to increased cell death and to a delay in mitosis (Edwards et al. 1974).

Interestingly, in our study, the negative impact of hyperthermia on cell proliferation seems to be partially compensated by the addition of HC to culture media. It has been proven that HC can stimulate DNA synthesis (Takigawa et al. 1988), mitosis in a combination with growth factors (Hoshi et al. 1982), and cellular growth and increase the lifespan (Macieira-Coelho 1966). In our study, fibroblasts were exposed to the temperature of 39 °C, which could cause a delay in mitosis resulting in decreased proliferation. With the addition of HC, the negative effect of elevated temperature could be suppressed by stimulating the proliferation of delayed mitotic cells. The protective interaction between elevated temperature and HC might be cell line, time, and dose specific, as has been proven for independent experiments either with HC or hyperthermia.

To conclude, our results indicate that the elevated temperature (39 °C) had an ambiguous effect. After long-term exposure, decreased fibroblast proliferation was observed. However, after short-term exposure, elevated temperature seemed to slightly enhance cell proliferation compared with the control (37 °C). The addition of HC at a concentration of 0.1 mg/ml increased proliferation of fibroblasts at both tested temperatures. Interestingly, the interaction of both factors, elevated temperature (39 °C) and the addition of HC (0.1 mg/ml), positively stimulated proliferation in comparison to fibroblasts cultured at 39 °C without HC addition after long-term exposure. This suggests a protective effect of HC on proliferation of cells at elevated temperature.

## Data Availability

Not applicable.
